# The birth of a giant: evolutionary insights into the origin of auxin responses in plants

**DOI:** 10.15252/embj.2022113018

**Published:** 2023-02-14

**Authors:** Vanessa Polet Carrillo‐Carrasco, Jorge Hernandez‐Garcia, Sumanth K Mutte, Dolf Weijers

**Affiliations:** ^1^ Laboratory of Biochemistry Wageningen University Wageningen the Netherlands

**Keywords:** auxin, ecology, evolution, hormone response, plant biology, Plant Biology

## Abstract

The plant signaling molecule auxin is present in multiple kingdoms of life. Since its discovery, a century of research has been focused on its action as a phytohormone. In land plants, auxin regulates growth and development through transcriptional and non‐transcriptional programs. Some of the molecular mechanisms underlying these responses are well understood, mainly in *Arabidopsis*. Recently, the availability of genomic and transcriptomic data of green lineages, together with phylogenetic inference, has provided the basis to reconstruct the evolutionary history of some components involved in auxin biology. In this review, we follow the evolutionary trajectory that allowed auxin to become the “giant” of plant biology by focusing on bryophytes and streptophyte algae. We consider auxin biosynthesis, transport, physiological, and molecular responses, as well as evidence supporting the role of auxin as a chemical messenger for communication within ecosystems. Finally, we emphasize that functional validation of predicted orthologs will shed light on the conserved properties of auxin biology among streptophytes.

## Graphical abstract

This review gives us an evolutionary perspective on auxin, a key regulator of plant growth and development.

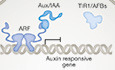

## Introduction

In search of the mysterious endogenous plant compound underlying differential growth, auxin was the first plant hormone to be identified (Darwin & Darwin, 1881; Went & Thimann, 1937). Early experiments showed that auxin acts as a chemical messenger by transporting a “growth stimulus” from shoots to roots upon the perception of light and gravity (Darwin & Darwin, 1881; Boysen‐Jensen, 1910). The chemical was identified as indole‐3‐acetic acid (IAA) and represents plants' most abundant natural auxin (Kögl *et al*, 1934; Went & Thimann, 1937). What is remarkable about auxin is that it influences almost every aspect of plant growth and development. The ability to act locally is conditioned by the ability of plants to control local synthesis, inactivation, and cell–cell transport (Friml, 2021). Auxin not only acts as a coordinating signal between cells, tissues, and organs, but also mediates interaction with the environment, both as a trigger of environmentally controlled development and as a messenger for interkingdom communication (Weijers & Wagner, 2016; Kunkel & Harper, 2018).

Most studies have focused on the role and influence of auxin in the growth and development of flowering plants (angiosperms). However, a similar range of activity has been reported in bryophytes (Reviewed in Kato *et al*, 2018), suggesting a conserved role across land plants. In addition, some extant green algae also show responses to exogenous IAA (Wood & Berliner, 1979; Ohtaka *et al*, 2017). Growing phylogenetic and molecular data indicate that the ancestor of modern land plants made their transition to land around 480 million years ago and evolved from streptophyte algae most closely related to the Zygnematophyceae; a group of filamentous and unicellular algae that live in terrestrial and freshwater environments (Wodniok *et al*, 2011; Timme *et al*, 2012; Hess *et al*, 2022).

**Figure 1:**
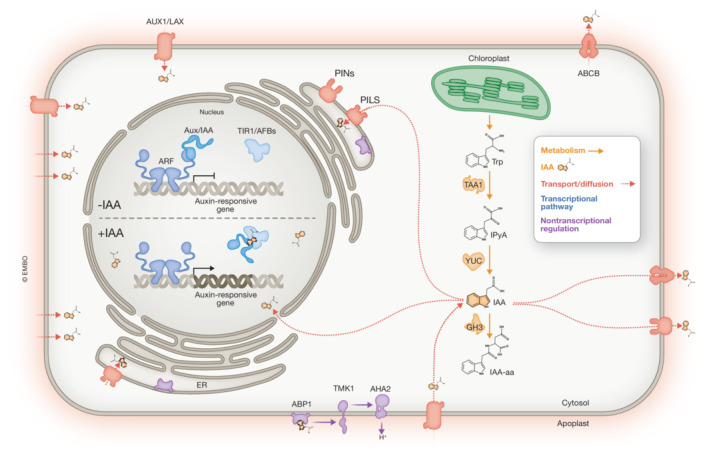
Illustration of a land plant cell with the main components of auxin biology The most common precursor of Indole 3‐acetic acid (IAA; indicated in orange structures) biosynthesis is tryptophan synthesized in the chloroplasts. In plants, indole‐3‐pyruvic acid (IPyA) is the major intermediate and this involves the function of TRYPTOPHAN AMINOTRANSFERASE OF ARABIDOPSIS (TAA) followed by decarboxylation catalyzed by members of the YUCCA (YUC) family. IAA can then be stored in the cells by forming amino acid conjugates catalyzed by GRETCHEN HAGEN3 (GH3) auxin‐amido synthetases. IAA is transported inside the cells via importers (AUX/LAX) or by diffusion (protonated form IAAH). IAA is an anionic form inside the cells and is exported via PIN proteins localized at the plasma membrane (PM). PILS is a family of proteins mainly localized to the endoplasmic reticulum (ER) and have an auxin‐transport function and presumably contribute to intracellular auxin homeostasis. A second family of PM transporters, ABCBs, act mainly as IAA efflux carriers, working together with PINs in transporting IAA outside the cells. Inside the nucleus, IAA regulates the NAP. When auxin is abundant in the nuclear environment (orange structures) it binds to members of the TIR1/AFB receptor family (light blue) part of a ubiquitin ligase complex and to AUX/IAA repressors (light blue), targeting the latter for degradation. Thus, the auxin response factors (ARFs) can activate the transcription of auxin‐responsive genes. AUXIN‐BINDING PROTEIN1 (ABP1) functions as auxin receptor in the apoplast, and it is known to mediate transmembrane kinase (TMK1) proteins that mediate rapid responses. TMK1 mediates the phosphorylation of AHA‐plasma membrane H^+^‐ATPases in the presence of auxin, which is translated into acidification of the apoplast.

Therefore, there is an increasing effort among the scientific community to understand which innovations allowed these photosynthetic organisms to move from water to land and become the dominant macroscopic flora we see around us today (de Vries & Archibald, 2018). Among such innovations, signaling pathways regulated by phytohormones have been proposed to be a main driver for the adaptation of plants to new niches and the evolution of complexity (Rensing, 2018; Blázquez *et al*, 2020). In this review, we address the evolutionary origins of auxin biology in plants (Fig 1). Given that the origins and early diversification of auxin functions predate and coincide with plant terrestrialization (Bowman *et al*, 2017; Mutte *et al*, 2018), we place particular emphasis on bryophytes and streptophyte algae. We consider endogenous auxin—IAA biosynthesis, transport, physiological and molecular responses, and the role of auxin in communication within different ecosystems. In doing so, we can now start to understand the evolutionary trajectory that has allowed auxin to become a signaling molecule that regulates so many biological processes in the plant kingdom—the regulatory “giant” of plant biology.

## Where the giants are…

Auxin is found in multiple kingdoms of life. IAA has been hypothesized to be synthesized in more than 80% of prokaryotes, including several phyla of archaea and bacteria (Zhang *et al*, 2019 †). Many of them are directly associated with plants with members that include pathogens, nitrogen‐fixing symbionts, plant growth‐promoting rhizobacteria, as well as non‐symbiotic bacteria and marine microorganisms (Amin *et al*, 2015; Cox *et al*, 2018; Kunkel & Harper, 2018). Auxins have also been found in several eukaryotes, such as fungi that form symbiotic or non‐symbiotic associations with green lineages (Cohen *et al*, 2002; Rao *et al*, 2010; Chanclud & Morel, 2016; Leontovyčová *et al*, 2020). Other eukaryotes, such as brown and red algae, as well as many chlorophytes and streptophytes, show endogenous accumulation of IAA (Basu *et al*, 2002; le Bail *et al*, 2010; Mikami *et al*, 2016; Bogaert *et al*, 2019). Given this extremely broad distribution, IAA accumulation in any organism should be interpreted cautiously, unless the presence of associated microbes can be excluded.

That there is no universal pathway for the biosynthesis of IAA (Morffy *et al*, 2020) is perhaps coupled to its structural simplicity. However, in most cases, tryptophan (Trp) acts as a precursor. The production of IAA flows through several enzymatic pathways named after their intermediate compounds, such as indoleacetamide (IAM) and indole‐3‐pyruvic acid (IPyA) (Reviewed in Morffy & Strader, 2020). Fungi and bacteria generally use the IAM and IPyA pathways for auxin production (Chanclud & Morel, 2016; Morffy *et al*, 2020). The non‐plant IPyA pathway is based first on the formation of indole‐3‐acetaldehyde (IAAld) by decarboxylases, followed by the conversion of IAAld to IAA by a dehydrogenase (Leontovyčová *et al*, 2020; Dong *et al*, 2022). In plants, IPyA is the major biosynthetic intermediate of IAA synthesis (Mashiguchi *et al*, 2011; Morffy *et al*, 2020). Here, the deamination of Trp to IpyA is catalyzed by the TRYPTOPHAN AMINOTRANSFERASE OF ARABIDOPSIS (TAA) family of aminotransferases (Tao *et al*, 2008; Mashiguchi *et al*, 2011). Subsequently, IPyA is decarboxylated to IAA by the YUCCA (YUC) family of flavin‐containing monooxygenases (FMO; Mashiguchi *et al*, 2011; Stepanova *et al*, 2011; Dai *et al*, 2013).

**Figure 2:**
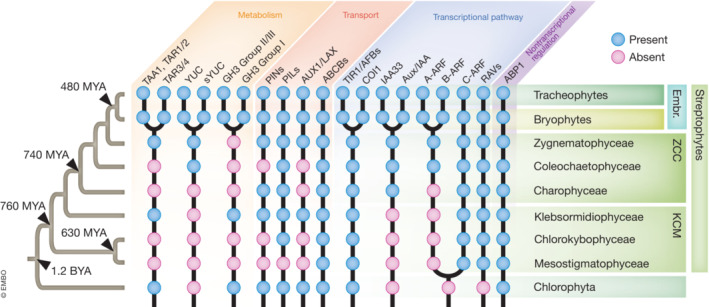
Schematic summary of presence or absence of ancestral copies of the main genetic components of auxin biology in plants Biosynthesis and metabolism (orange), transport (pink), transcriptional pathway (light blue), and non‐transcriptional regulation (purple). Orthologs present (blue circles) or absent (pink circles). Land plants also referred to as embryophytes (Embr.) are divided into tracheophytes and bryophytes and belong to the Viridiplantae (plants) clade of eukaryotic organisms. Plants are found in almost every habitat on Earth and are divided into two main clades, streptophytes and chlorophytes. The Streptophytes comprise a group of green algae, the streptophyte algae, and the land plants (One Thousand Plant Transcriptomes Initiative, 2019). Streptophyte algae are a paraphyletic group of extant algae that encompass the lower‐branching KCM‐grade classes, Klebsormidiophyceae, Chlorokybophyceae, and Mesostigmatophyceae and the higher‐branching ZCC‐grade classes, Zygnematophyceae, Coleochaetophyceae, and Charophyceae (de Vries & Archibald, 2018).

Phylogenetic analyses have shown that both TAA and YUC gene families are deeply conserved across genomes of land plants, being represented both in tracheophytes (lycophytes, ferns, gymnosperms and angiosperms) and bryophytes (liverworts, hornworts, and mosses; Fig 2; Yue *et al*, 2014; Bowman *et al*, 2017). The conserved role of the IAA biosynthetic pathway in land plants has been established through genetic and pharmacological analyses in bryophytes (Landberg *et al*, 2013; Eklund *et al*, 2015). For example, in the liverwort *Marchantia polymorpha*, disruption of this pathway causes dramatic developmental defects (Eklund *et al*, 2015).

**Figure EV1:**
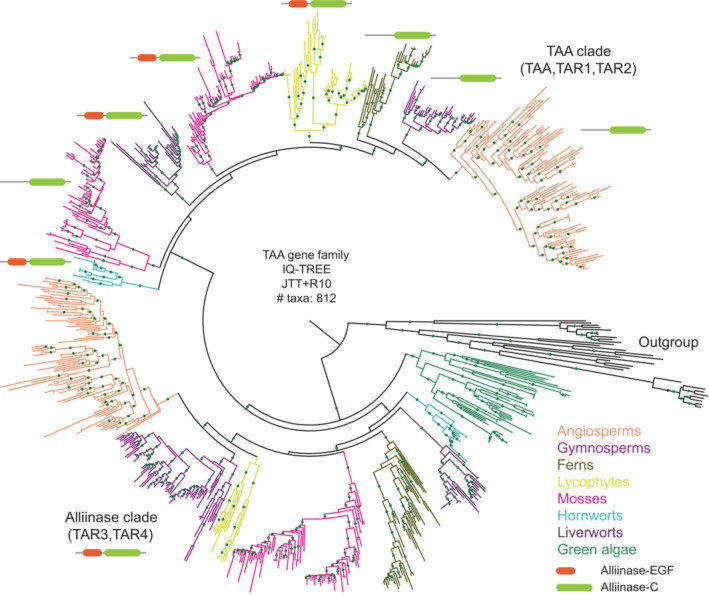
Phylogenetic tree of the TAA gene family with green algae and land plant homologs Protein domains, Alliinase‐EGF and Alliinase‐C are indicated with “red” and “green” representations, respectively. TAR3/TAR4 clade shows the consistent presence of both domains in all lineages, whereas in TAA/TAR1/TAR2 clade, some phyla lack Alliinase‐EGF domain. Aminotransferases from land plants, other than TAA members, were used as outgroup sequences to root the tree. Branches that are well supported (bootstrap > 75) are indicated by green dots. Orthologs from each phylum are represented in different colors, as indicated in the right bottom right legend. Basic information about the tree construction: “software,” “model of evolution,” and the “number of taxa” used for phylogenetic tree construction are indicated at the center. The complete tree can be found at the interactive Tree of Life (iTOL) repository: https://itol.embl.de/shared/dolfweijers.

The TAA gene family is divided into two clades that encode alliinase domain‐containing proteins (Figs 2 and EV1; Wang *et al*, 2014; Bowman *et al*, 2017). The TAA clade in *Arabidopsis* includes three homologs that contain an Alliinase‐C domain (IPR006948): TAA1, TAR1, and TAR2; the Alliinase clade includes two homologs, TAR3 and TAR4, which contain both an Alliinase‐EGF (Epidermal growth factor, IPR006947) domain at their N‐terminus and an Alliinase‐C domain at their C‐terminus. In embryophytes, the TAA subclade contains the orthologs that are involved in the biosynthesis of IAA, whereas the functions of orthologs that contain both Alliinase domains are yet to be described (Tao *et al*, 2008; Eklund *et al*, 2015). In this review, we extend previous analyses on the evolutionary origin of TAA homologs to the green algae. We have identified only one ortholog in each algal species (Fig EV1). Most of the chlorophyte orthologs have only the Alliinase‐C domain. In contrast, most of the orthologs found in streptophyte algae contain both the Alliinase‐EGF and the Alliinase‐C domains. These data suggest that the TAA/Alliinase homologs that are present in streptophyte algae are reminiscent of an ancestral gene with unknown substrate specificity present in the last common land plant/bryophyte ancestor. This gene is likely to have duplicated and neofunctionalized in land plants during evolution (Bowman *et al*, 2021).

**Figure EV2:**
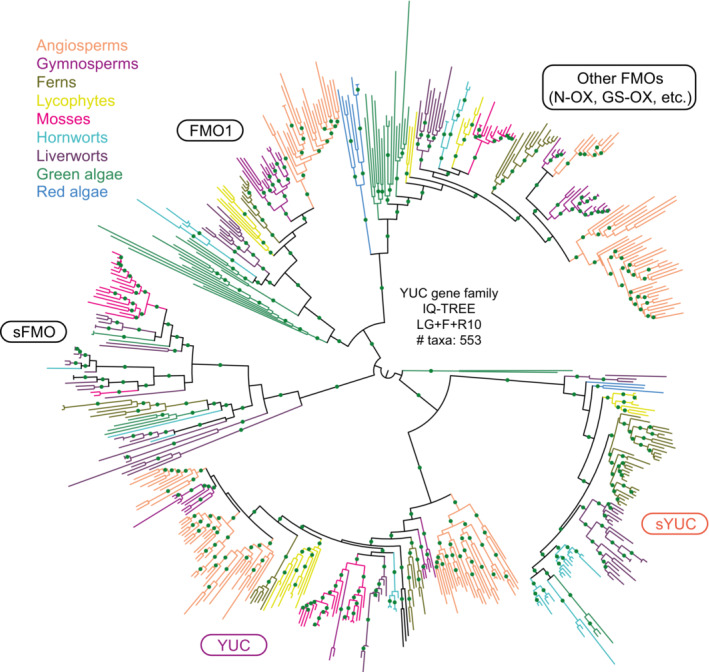
Phylogenetic tree of the YUC gene family with algae and land plant homologs The different FMO clades are indicated by representative *Arabidopsis* family member names (FMO, flavin monooxyenase 1; GS‐OX, FMO glucosinolate S‐oxygenase; N‐OX, FMO N‐oxygenases; YUC, Yucca). Branches that are well supported (bootstrap > 75) are indicated by green dots. Orthologs from each phylum are represented in different colors, as indicated in the bottom right legend. Basic information about the tree construction: “software,” “model of evolution,” and the “number of taxa” used for phylogenetic tree construction are indicated at the center. The complete tree can be found at the interactive Tree of Life (iTOL) repository: https://itol.embl.de/shared/dolfweijers.

The *YUC* gene family in land plants is divided into two clades (Figs 2 and EV2; Wang *et al*, 2014). The YUC clade contains 11 members in *Arabidopsis*. Some other YUCs from embryophytes are placed in a sister clade to the YUC (sYUC, Fig EV2), but this clade seems to have been independently lost in seed plants and mosses. Some green algae sequences form a sister clade basal to both YUC and sYUC. Further phylogenetic analysis including other FMO family proteins such as flavin monooxygenase 1 (FMO1) and FMO glucosinolate S‐oxygenase (GS‐OX) showed that YUC orthologs in land plants and in streptophyte algae are different from other FMO orthologs (Fig EV2). Both clades of YUCs are conserved among land plants but are not common to all members of the green algae ZCC clade (Zygnematophyceae, Coleochaetophyceae, and Charophyceae; Fig 2). Other phylogenetic studies hypothesize that, even though YUC homologs are identified in algal lineages, these FMOs enzymes are likely an innovation of land plants acquired by horizontal gene transfer from bacteria (Yue *et al*, 2014; Bowman *et al*, 2017). Hence, the origin of this gene family in streptophyte algae remains unsolved. Cross‐species complementation, biochemical, and genetic studies in model streptophyte algae are needed to validate the conservation of an ancestral function.

**Figure EV3:**
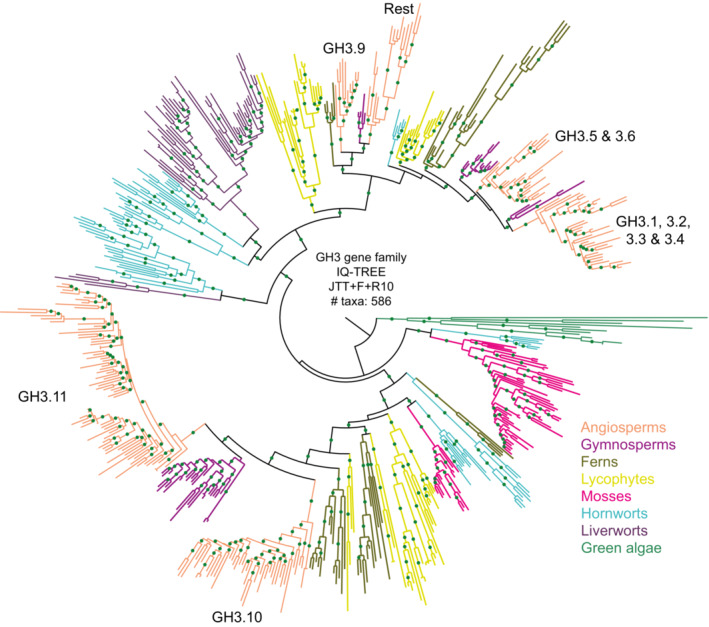
Phylogenetic tree of the GH3 gene family with green algae and land plant homologs Respective *Arabidopsis* orthologs that are present in the specific clade are mentioned with the corresponding *Arabidopsis* family member names. “Rest” includes GH3.7, 3.8, 3.12 until 3.19. Branches that are well supported (bootstrap > 75) are indicated by green dots. Orthologs from each phylum are represented in different colors, as indicated in the bottom right legend. Basic information about the tree construction: “software,” “model of evolution,” and the “number of taxa” used for phylogenetic tree construction are indicated at the center. The complete tree can be found at the interactive Tree of Life (iTOL) repository: https://itol.embl.de/shared/dolfweijers.

Besides synthesis, other mechanisms control the amount of IAA available during plant growth. These include transport (see next section), conjugation, glycosylation, and oxidation (Reviewed in Casanova‐Sáez *et al*, 2021). One of the most common inactivation forms of IAA is the formation of amino acid conjugates catalyzed by GRETCHEN HAGEN3 (GH3) auxin‐amido synthetases (Staswick *et al*, 2005; Ludwig‐Müller *et al*, 2009). Some conjugates such as IAA‐Ala or IAA‐Leu are reversibly converted to free IAA by amidohydrolases and are considered auxin storage forms (LeClere *et al*, 2002; Rampey *et al*, 2004). While others such as IAA‐Asp and IAA‐Glu are irreversibly converted to free IAA and are considered auxin catabolites (Rampey *et al*, 2004; Mellor *et al*, 2016; Casanova‐Sáez *et al*, 2021). Phylogenetic and metabolomic analyses indicate that members of the GH3 family and amino acid‐IAA conjugates are common to land plants (Staswick *et al*, 2005; Terol *et al*, 2006; Bowman *et al*, 2021). The GH3 family is divided into three phylogenetic subgroups with different substrate preferences (Figs 2 and EV3; Bowman *et al*, 2021). In angiosperms, members of group II function in auxin conjugation, while those of group I and III use jasmonic acid (JA) or benzoate as substrates, respectively (Staswick *et al*, 2005; Okrent *et al*, 2009). In the moss *Physcomitrium patens*, two GH3 homologs of group I have been shown to convert IAA and JA to amino acid conjugates, suggesting a conserved function of this gene family in the land plants (Ludwig‐Müller *et al*, 2009). All the groups seem to have emerged from a single streptophyte algae ancestral GH3 gene with unknown substrate specificity as observed in a known GH3 homolog of *Klebsormidium nitens* (KCM clade, Klebsormidiophyceae, Chlorokybophyceae, and Mesostigmatophyceae). However, as this homolog cannot be assigned to any specific subgroup, it has been hypothesized that *K. nitens* GH3 could have been obtained from bacteria by horizontal gene transfer (Bowman *et al*, 2021). In this scenario, the molecular machinery by which IAA‐amino acid conjugation maintains auxin homeostasis would be a land plant innovation. Thus genomic, and biochemical data are ambivalent with regard to the capacity of streptophyte algae to synthesize or inactivate auxin in a similar way to land plants.

## Moving giants…

Studies conducted in vascular plants show that once auxin is synthesized in source organs, such as young leaves, it is transported over both short and long distances, partly by diffusion and partly through carriers that offer directionality. The “polarity” of auxin distribution is mainly attributed to the asymmetric localization of members of the PIN‐FORMED (PIN) family of auxin efflux carriers at the plasma membrane (Bennett *et al*, 1996; Chen *et al*, 1998; Gälweiler *et al*, 1998). Therefore, the localization of PIN proteins establishes the directionality of auxin flow, known as “polar auxin transport,” as reviewed in Michniewicz *et al* (2007). Phylogenetic analyses show that PIN proteins are conserved along the green lineages (Fig 2) and have been classified into canonical and non‐canonical based on the length of their central hydrophilic loop and their cellular localization (Billou *et al*, 2005; Reviewed in Bennett, 2015; Hammes *et al*, 2022). Genetic disruption of canonical PINs in *P. patens* affects auxin transport and tropisms in gametophores, similar to the effects of auxin transport disruption in *Arabidopsis* shoot and root tropisms (Bennett *et al*, 2014). In streptophyte algae, homologs of PIN proteins form a single clade (Bogaert *et al*, 2022). Functional studies of a *K. flaccidum* PIN show that this protein acts as an efflux carrier in an angiosperm heterologous system (Skokan *et al*, 2019), suggesting a conserved origin of the biochemical properties of PINs in streptophyte algae (Boot *et al*, 2012; Skokan *et al*, 2019). However, genetic dissection of PIN function in model green algae is necessary to validate the transport and biological functions of these proteins.

Another protein family of auxin transporters, the PILS (PIN‐LIKES), has been identified based on homology to the PINs. These proteins are localized to the endoplasmic reticulum, and genetic and pharmacological analyses indicate that they regulate intracellular auxin homeostasis in *Arabidopsis* (Barbez *et al*, 2012). Interestingly, phylogenetic analyses of the PILS family show that its members are present in almost all eukaryotic clades, including all the green lineages (Bogaert *et al*, 2022). These data suggest that if the PILS‐mediated transport function is auxin‐specific, intracellular auxin regulation may have predated the intercellular directional auxin transport (Bogaert *et al*, 2022). A key question is whether such an ancient function in intracellular partitioning served a role in physiological regulation and signaling, or rather in detoxification or metabolism.

Another family of proteins that acts as auxin efflux carriers in angiosperms is the ATP‐binding cassette subfamily B/multidrug resistance/phosphoglycoprotein (ABCB/MDR/PGP; Noh *et al*, 2001; Verrier *et al*, 2008), with some homologs showing auxin influx properties (Kamimoto *et al*, 2012; Reviewed in Hammes *et al*, 2022). However, the major contribution to active IAA uptake in plants is made by members of the AUXIN RESISTANT 1/LIKE AUXIN RESISTANT 1 (AUX1/LAX) influx carriers (Bennett *et al*, 1996). This family of plasma membrane proton co‐transporters was first identified in *Arabidopsis* auxin‐resistant *aux* mutants. Members of the ABCB and AUX1/LAX carrier families are deeply conserved throughout green lineages (Fig 2; Vosolsobě *et al*, 2020; Bowman *et al*, 2021). Taken together, while there are either well‐defined or more distantly related homologs of all auxin transporters across the green kingdom, functional studies for most streptophytes, that would confirm the conservation of auxin transport driven by PIN, PILS, ABCB, and AUX1/LAX carriers, are lacking.

A picture emerges in which auxin occurs broadly, with land plants (and possibly some algae) actively controlling its accumulation. But what responses occur? What mechanisms underlie these? And how did these originate and evolve?

## How to respond to giants…

Auxin controls almost every process in the growth and development of plants, and there is a considerable insight into the physiological, cellular, biochemical, and genetic mechanisms triggering these responses (Weijers & Wagner, 2016; Leyser, 2018). Both endogenous and externally applied auxin triggers changes in growth (Reviewed in Cooke *et al*, 2002). For example, auxin causes responses such as inducing cell elongation in *Arabidopsis* hypocotyls that translate to overall hypocotyl growth (Lin *et al*, 2021). The same auxin stimulus, however, leads to the growth inhibition of roots, a process underlying gravitropic bending (Evans *et al*, 1994; Fendrych *et al*, 2018). These growth differences between organs suggest specific mechanisms for auxin‐dependent growth regulation, which has triggered the curiosity of researchers for many decades (See next section).

In the green alga *Chara*, auxin has a stimulatory effect on the development of rhizoids, similar to its effects on bryophytes, and the induction of root hairs in angiosperms (Sandan & Ogura, 1957; Klambt *et al*, 1992; Jones & Dolan, 2012). In land plants, the development of rooting systems is hypothesized to be mediated by a conserved auxin—RSL (ROOT HAIR DEFECTIVE 6‐LIKE) genetic network (Jones & Dolan, 2012). Other examples of auxin responses in streptophyte algae have been observed with exogenous IAA treatments of *Micrasterias thomasiana* and *K. nitens*. Here, auxin either promotes or inhibits cell division, which could be partly explained by the large differences in the IAA concentrations used for the treatments (Wood & Berliner, 1979; Ohtaka *et al*, 2017). Thus, while there are physiological effects, it is unclear whether there is a generic algal auxin response mechanism.

In addition to effects on growth, several reports document auxin responses at a cellular level that include plasma membrane depolarization, cytosolic calcium influx, reorganization of the actin network, acidification/alkalinization of the apoplast, and stimulation of cytoplasmic streaming, sometimes within seconds or minutes of auxin application (Thimann & Sweeney, 1937; Arieti & Staiger, 2020; Friml *et al*, 2022; Li *et al*, 2022). In *Nitella*, a charophycean alga, auxin has been shown to stimulate cytoplasmic streaming of internodal cells in a similar way to that seen in *Arabidopsis* root epidermal cells (Thimann & Sweeney, 1937; Friml *et al*, 2022). A key question is what biological functions of auxin are mediated by this array of fast and slow cellular responses across the plant kingdom.

## Giant steps…

Essentially, all molecular mechanisms that are known to mediate auxin responses were identified in angiosperms, chiefly in *Arabidopsis*. The auxin response involves a relatively well‐understood transcriptional branch—the nuclear auxin pathway (NAP)—(Reviewed in Weijers & Wagner, 2016; Leyser, 2018). There is also a branch of rapid auxin responses that may mediate such fast cellular responses that cannot be mediated by transcriptional changes (See below; Reviewed in Dubey *et al*, 2021).

The NAP has been a subject of intense investigation for the last three decades (Abel *et al*, 1994; Ulmasov *et al*, 1997a, 1997b, 1999; Boer *et al*, 2014; Kato *et al*, 2020), revealing a transcriptional module controlled by auxin that is likely only to be present in land plants (Martin‐Arevalillo *et al*, 2019; Bowman *et al*, 2021). Forward genetic screenings using *Arabidopsis* root growth inhibition have been invaluable in identifying the key components in auxin response (Abel *et al*, 1995). The NAP involves the perception of auxin by transport inhibitor response 1/auxin F‐box (TIR1/AFB) proteins (Kepinski & Leyser, 2005), substrate‐binding subunits of ubiquitin ligases (Tan *et al*, 2007). When cellular IAA levels rise, the hormone acts as a molecular glue that increases the affinity of TIR1/AFB to its target substrates, the Aux/IAA transcriptional inhibitor proteins (Tan *et al*, 2007). This interaction triggers subsequent ubiquitin‐dependent degradation of the repressors (Gray *et al*, 2001) and presumably enhances the production of cAMP catalyzed by an adenylate cyclase embedded in the auxin pocket of TIR1/AFBs (Qi *et al*, 2022). At low auxin concentrations, Aux/IAAs recruit TOPLESS co‐repressors (Szemenyei *et al*, 2008), bind to, and inhibit the activity of DNA‐binding ARF transcription factors (Korasick *et al*, 2014; Nanao *et al*, 2014). Thus, auxin regulates this system by promoting proteolysis of Aux/IAAs and releasing ARFs from inhibition, leading to changes in the expression of hundreds to thousands of genes (Weijers & Wagner, 2016). Although the model is simple, in most land plants, each gene family has multiple paralogs resulting in an extensive combinatorial network with multiple outputs (Finet *et al*, 2013; Freire‐Rios *et al*, 2020).

**Figure 3:**
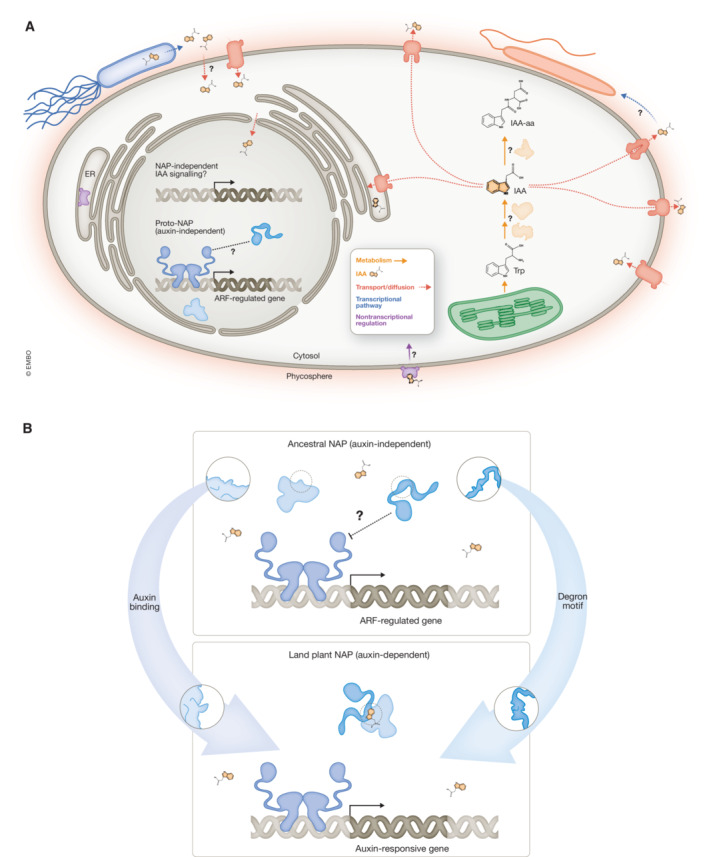
Evolutionary scenario of ancestral auxin biology in streptophytes (A) Auxin (IAA) has been found in extant streptophyte algae and likely in the last common ancestor of streptophytes. However, the biosynthetic pathway for its production remains unknown. In streptophyte algae, there are predicted orthologs of gene families that in plants catalyze the conversion of Trp to IAA via IPyA, TAA, and YUC. Among the mechanisms that contribute to regulating IAA levels in plants, storage mediated by members of the GH3 gene family remains controversial in algae as only a few members of this family have been reported. Regarding transport (red), predicted orthologs of PIN, PILS, ABCB, and AUX/LAX are present in members of the green algae, suggesting that the transport function was present in an ancestral streptophyte. Several responses to auxin have been observed in streptophyte algae, but the mechanisms underlying these responses are unknown. In land plants, fast responses to auxin are mediated by auxin perception via the ABP1‐TMK1 module. In extant algae, the ABP1 gene family is well conserved, suggesting a plausible scenario in which an ancestral streptophyte might have been responding to local auxin produced in the phycosphere (red). (B) Homologs of the three main components of the NAP are present in extant algae. However, the TIR1‐like receptor and Aux/IAA‐like predicted orthologs lack the components to interact with auxin. Therefore, there is a plausible land plant scenario in which a diversified Proto‐ARF network came to be regulated by auxin upon innovations in TIR1 and Aux/IAA protein families.

Auxin responses regulated by the NAP are deeply conserved across land plants, as shown by different phylogenetic, transcriptomic, functional, and biochemical analyses (Flores‐Sandoval *et al*, 2015; Kato *et al*, 2015, 2020; Mutte *et al*, 2018; Fig 2). Recent genetic studies show that the NAP controls the development of bryophytes such as the liverwort *M. polymorpha* and the moss *P. paten*s in a manner similar to its action in angiosperms (Fig 2; Lavy *et al*, 2016; Reviewed in Kato *et al*, 2018). Following work in *Physcomitrium* (Prigge *et al*, 2010; Lavy *et al*, 2016), genetic and structural analyses in *Marchantia* revealed a deeply conserved function of a minimal set of auxin signaling components (Reviewed in Kato *et al*, 2018; Kato *et al*, 2020). This bryophyte contains one gene copy of each subclass within the ARF family including the canonical A, B, and C types, a non‐canonical ARF (subclass lacking DNA‐binding domain), and a single Aux/IAA and TIR1 proteins (Flores‐Sandoval *et al*, 2015; Kato *et al*, 2015; Mutte *et al*, 2018). Genetic disruption of the A‐class ARF (MpARF1) causes severe developmental phenotypes and a lack of auxin responses that cannot be complemented with a different class of ARF (MpARF2 or MpARF3; Kato *et al*, 2020). These data suggest dedicated functions of each ARF class. Domain swapping complementation experiments using the Mp*arf1* mutant and the domains of all the ARF classes showed that class‐A and ‐B compete for the same DNA‐binding sites while class‐C ARF is not involved in auxin responses (Kato *et al*, 2020). Class‐A ARF can recruit MpIAA and activate auxin‐dependent gene expression, while Class‐B ARF is not auxin‐dependent (Kato *et al*, 2015, 2020). In the current model, the expression patterns and stoichiometries of both proteins determine the responsiveness of cells to auxin (Kato *et al*, 2020). This simple model may explain the molecular basis of the transcriptional auxin response network in land plants. It does not, however, tell us how this transcriptional machinery evolved. Of the three NAP protein families, streptophyte algae only contain well‐defined ARF‐like proteins (proto‐ARFs) with predicted similar architecture and perhaps biochemical function to land plant ARFs (Fig 2; Mutte *et al*, 2018; Martin‐Arevalillo *et al*, 2019). Phylogenetic analysis and prediction of Proto‐ARF structures show that they fall into two clades: A/B and C, which likely represent the ancestral precursors of land plant ARFs and that already diverged in an ancient clade of streptophyte algae (Fig 2). Accordingly, recent biochemical analysis shows that a Proto‐C‐ARF from *Chlorokybus atmophyticus* has a DNA‐binding specificity that differs from land plants A and B, and that has been maintained in class‐C ARFs along evolution (Martin‐Arevalillo *et al*, 2019). Presence of Proto‐C ARFs is common along the streptophyte algae with no specific pattern of appearance or retention, while Proto A/B‐ARFs have been found mainly in the ZCC clade (Martin‐Arevalillo *et al*, 2019; Mutte, 2020). These observations indicate that an ancestral Proto‐C ARF could have duplicated and possibly acquired new functions in the common ancestor of the ZCC algal clade and the land plants (Fig 2; Martin‐Arevalillo *et al*, 2019). Curiously, most of the reported species in this algal clade contain only one class of Proto‐ARF, which may reflect independent losses or limited depth of sequencing of the sampled species. The genomes of streptophyte algae encode predicted TIR1/AFB‐like and AUX/IAA‐like proteins (Bowman *et al*, 2017; Mutte *et al*, 2018; Fig 2). However, in both cases, they lack critical residues to interact with auxin. Phylogenetic analysis of *Aux/IAA* homologs shows that this gene family is deeply conserved in the embryophytes, divided into two clades, canonical and non‐canonical (Fig 2; Mutte *et al*, 2018). In streptophyte algae, there are predicted Aux/IAA orthologs only in the ZCC clade, suggesting that this Proto‐AUX/IAA represents the ancestral precursor of the neofunctionalized land plant AUX/IAAs (Bowman *et al*, 2017; Mutte *et al*, 2018). The overall predicted structures of TIR1‐like proteins are similar to land plant auxin (and jasmonate) receptors, but amino acids for auxin binding are not conserved. Taken together, the available functional and phylogenetic analysis allows a plausible ancestral land plant evolutionary scenario to be proposed, in which a diversified Proto‐ARF network was coopted for regulation by auxin upon the evolution of an increased auxin‐binding affinity in proto‐TIR1 and proto‐Aux/IAA proteins (Fig 3). Thus, phylogenomic data suggest that the transcriptional auxin signaling pathway is most likely a land plant innovation. This begs the question of what the ancestral function of the proto‐ARF factors is, and what mutational trajectory ushered the birth of the auxin response system. Functional studies in emerging genetic models of extant streptophyte algae should help answer these questions (Zhou *et al*, 2020). Likewise, reconstructing ancestral proteins could help to understand the evolutionary trajectory that caused the auxin responses, protein structure, and function to diverge in the green lineages (Scossa & Fernie, 2021).

Some auxin responses, such as the rapid root growth inhibition in Arabidopsis, happen too rapidly to involve transcription, yet are also dependent on AFB1 function (Dindas *et al*, 2018; Fendrych *et al*, 2018; Serre *et al*, 2021). Components of a non‐transcriptional AFB1 pathway remain unknown, and it is unclear whether such functions are shared by other species and other TIR1/AFB receptors (Prigge *et al*, 2020). Recent studies show that auxin triggers very rapid (< 2 min) phosphorylation of about a thousand proteins in Arabidopsis roots, and that this requires both the auxin‐binding protein 1 (ABP1) and its potential partner protein transmembrane kinase 1 (TMK1; Friml *et al*, 2022). Outcomes of this rapid signaling appear to include the activation of H+‐ATPases that acidify the apoplast and the regulation of Myosin XI that may influence cytoplasmic streaming (preprint: Han *et al*, 2021; Lin *et al*, 2021; Friml *et al*, 2022). Interestingly, disruption of ABP1 and TMK leads to defects in both regeneration from callus and regeneration of vascular strands around a stem wound (preprint: Han *et al*, 2021; Friml *et al*, 2022). However, it remains unknown how the ultrafast auxin phospho‐response is connected to slower, developmental responses. Important questions are whether these fast responses are conserved across species. If so, are they regulated by the ABP1‐TMK module in all the land plants (and perhaps algae)? ABP1 is a well‐conserved gene family that is maintained as a single copy in all the green lineages including all the streptophyte algae lineages (Fig 2). Clearly, there are new twists and turns ahead in the adventurous life of the auxin giant…

## Communicating through giants…

Auxin produced by microbes has been shown to trigger responses in plants, including changes in growth patterns or developmental programs (Sukumar *et al*, 2012). In some cases, these changes can be linked to the establishment of symbiosis, such as rhizobia‐associated nodule formation (Hussain & Hasnain, 2011); in other cases, pathogens induce growth changes—for example, rhizogenesis during *Agrobacterium rhizogenes* infections (Falasca *et al*, 2000). Auxin can act as an immunity suppressor, mainly through antagonizing the salicylic acid response (Kunkel & Harper, 2018). Other effects seem to be a direct outcome of the established partnership. For example, root architecture changes or overall plant growth can be linked to endogenous auxin signaling modulation. These effects have been commonly reported in the last decades and have been thoroughly reviewed elsewhere for either pathogenic or mutualistic symbioses (Boivin *et al*, 2016; Kunkel & Harper, 2018).

While microbes widely use auxins to modulate plant development, they themselves can also respond to the presence of auxin. Some studies have reported changes in microbe pathogenicity or colonization habits in response to auxins. For example, just as mutant strains of *Fusarium oxysporum* with enhanced IAA production resulting from overexpression of auxin biosynthetic genes (tryptophan‐2‐monooxygenase and indole‐3‐acetamide hydrolase) show enhanced virulence of the fungi to *Orobanche spp*. (broomrapes; Cohen *et al*, 2002), mutant strains of the fungi *Hebeloma cylindrosporum* show an enhanced ability to establish mycorrhizal associations with pines (Tranvan *et al*, 2000). This has also been reported for bacteria such as *Rhizobium etli* (Spaepen, 2015). In the case of the phytopathogenic bacteria *Pseudomonas syringae*, auxin production leads not only to increased bacterial growth itself but also to defense inhibition and changes in plant development (Djami‐Tchatchou *et al*, 2020). These and other studies support the idea of auxin acting as a communication signal between plants and microbes in the rhizosphere (Spaepen, 2015). However, many of these effects can be directly attributed to microbe‐produced IAA; much less attention has been paid to the role of plant‐derived auxins in the rhizosphere. Nonetheless, there are some indications that microbes sense and use plant‐derived IAA during symbiosis initiation. For example, *Lotus japonicum* root hairs and epidermal cells show increased IAA production prior to mycorrhiza formation (Nadzieja *et al*, 2018), which is important for the association with the fungal symbiont, suggesting that plant IAA is part of the chemical dialog involved in this symbiosis (van Noorden *et al*, 2006; Nadzieja *et al*, 2018).

While numerous studies have shown auxin being used for interkingdom communication between plants and microbes, there are also pieces of evidence pointing to auxin performing an endogenous role in microbes unrelated to plant interactions. Several studies indicate that prokaryotes manifest clear responses to IAA. For example, transcriptional profiling after IAA treatment in *Escherichia coli*, *Agrobacterium tumefaciens*, or *Bradyrhizobium japonicum* shows a general shift towards stress response activation (Bianco *et al*, 2006; Yuan *et al*, 2008; Donati *et al*, 2013). Some of these responses may derive from co‐evolution with plant hosts: for example, *Azospirillum brasilense* shows extensive transcriptional changes after IAA addition, of which some can be linked to the establishment of bacterium–plant interactions (van Puyvelde *et al*, 2011). Yet, these responses may also be part of a more general metabolic homeostasis system, which would agree with the idea of IAA being used as a carbon source for bacteria (Jensen *et al*, 1995; Leveau & Lindow, 2005; Donoso *et al*, 2016; Conway *et al*, 2022). In any case, both possibilities are not mutually exclusive, and the widespread presence of IAA‐producing bacteria that are able to respond to its presence suggests that auxin is used as an interkingdom communication signal and may be a derived trait coopted from a commonly used endogenous metabolic signal.

Auxin action is not limited to unicellular or prokaryotic microbes, as it also triggers endogenous responses in fungi, including stress tolerance promotion (Fu *et al*, 2015; Hsiung *et al*, 2022). Other effects are the induction of pseudohyphal and hyphal growth in *Saccharomyces cerevisia*e and *Candida albicans*, respectively, both capable of IAA production but also of exogenous IAA active incorporation (Prusty *et al*, 2004; Rao *et al*, 2010). Morphogenic changes in dimorphic fungi, as well as the induction of stress tolerance mechanisms in yeasts, could be linked to competitive, defensive, or invasive behaviors, suggesting that IAA may be used as a neighboring cell detection signal in these organisms. Furthermore, fungi have been proposed to use IAA as a communication signal not only in plant‐fungal symbioses but also in fungal–fungal and fungi microalgae associations (Fu *et al*, 2015).

A key question is whether interkingdom auxin signaling is a shared property within the plant kingdom. Green algae–microbe interactions are well‐known in different ecological niches. For example, lichens, the alga–microbe symbioses, are known to produce auxins, mostly by the mycobiont (Epstein & Miles, 1967; Pichler *et al*, 2020). Auxins also seem positively to affect the growth of both fungi and algae, but the physiological meaning of these responses is unknown (Wang *et al*, 2010; Pichler *et al*, 2020). Free‐living chlorophyte algae can also perceive and respond to environmental auxins produced by nearby microbes, as has been shown for the unicellular algae *Chlorella vulgaris* (Trebouxiophyceae) and *Scenodesmus* sp. (Chlorophyceae; Bagwell *et al*, 2014).

While the molecular basis of free‐living green algae interactions with microbes has not been studied in depth, other eukaryotic algae have been shown to either use or respond to IAA present in their phycospheres (Amin *et al*, 2015; Labeeuw *et al*, 2016). This is the case of the cosmopolitan diatom *Pseudo‐nitzschia multiseries*, which associates with bacteria from the *Sulfitobacter* genus (Amin *et al*, 2015). The symbiosis formed involves bacterial IAA being uptaken by the alga, and Trp being released by it, and taken by the bacteria, forming an interspecies positive feedback loop dedicated to maintaining the association. Cells of *P. multiseries* are incapable of producing IAA but are able to respond to it, enhancing their growth and Trp production. Other nitrogenous compounds are also part of the metabolic exchange, but IAA and Trp seem, at least in part, to underlie the chemical communication necessary for the establishment and maintenance of the association. This example indicates that IAA can be used as a chemical signal in open water environments. Moreover, it pinpoints the importance of the phycosphere in enabling the exchange of molecules between neighboring cells, given that local concentrations created in this environment need to be significantly higher than bulk concentrations in water columns to be sensed and recognized by neighboring cells (Jonsson *et al*, 2009). Green algal microbiomes have recently been reported (Krohn‐Molt *et al*, 2017; Durán *et al*, 2022). While these microbes may be different from those of diatoms, their phycosphere does contain auxin‐producing bacteria. Moreover, green algae and land plants share at least a core of their microbiota (Durán *et al*, 2022), suggesting that a conserved core microbiome exists in the Viridiplantae. Among these, there are several auxin‐producing orders of bacteria. In summary, auxins seem to take part in the interkingdom communication occurring in very different ecosystems, including either land or aquatic environments.

## Conclusions and future perspectives

In recent years, transcriptomic and genome‐based phylogenetic inference has opened the door to predict and possibly reconstruct the evolutionary history of the ancestral components of auxin biology (Bowman *et al*, 2017; Mutte *et al*, 2018; Fig 2). However, more functional validations of the predicted extant orthologs are required to confirm the conservation of the biochemical properties of all the components and the conservation of the biological role of the components using emerging streptophyte genetic models (Delaux *et al*, 2019; Martin‐Arevalillo *et al*, 2019; Skokan *et al*, 2019; Zhang *et al*, 2020; Zhou *et al*, 2020; Kawai *et al*, 2022).

The evolution of the auxin signaling pathway in land plants results from a co‐adaptation of a pre‐existing transcriptional machinery to respond to a chemical signal, auxin (Fig 3B). Functional studies in streptophyte algae are required to understand which features were regulated by the ancestral algae transcriptional network that gave rise to the land plants. Comparative studies will also be instrumental to unravel the contribution of transcriptional responses to auxin on the diversity of extant green lineages.

Given that auxin is a molecule widespread among many kingdoms of life, several aspects of auxin biology must be considered in the light of evolution and ecology. Here we reviewed evidence suggesting that auxin acts as a cue to support symbiotic partnerships between green lineages, fungi, and bacteria. These data support a plausible evolutionary scenario in which external auxin (in the phycosphere) may have long acted as a chemical messenger for local cellular responses (Fig 3), followed by a later consolidation of endogenous auxin responses that control plant growth and development. Further dissection of auxin responses in algal models, deepening of the understanding of fast, non‐transcriptional auxin responses, and focus on ecological interactions among plants (in the broadest sense) and microbes will help illuminate the natural history of the auxin giant.

## Author contributions

**Vanessa Polet Carrillo Carrasco:** Conceptualization; writing – original draft; writing – review and editing. **Jorge Hernandez‐Garcia:** Funding acquisition; visualization; writing – review and editing. **Sumanth K Mutte:** Resources; formal analysis; writing – original draft; writing – review and editing. **Dolf Weijers:** Conceptualization; supervision; funding acquisition; project administration; writing – review and editing.

## Disclosure and competing interests statement

The authors have no competing interests to disclose.

## Supporting information



Expanded View Figures PDF

PDF+

## Footnotes

^†^
Correction added on 15 March 2023, after first online publication: This reference callout has been corrected.
