# Supplementary material for: The birth of a giant: evolutionary insights into the origin of auxin responses in plants
Source: EMBO J. 2023 Feb 14;42(6):e113018. doi: 10.15252/embj.2022113018 (PMC10015382; doi:10.15252/embj.2022113018)
Supplement: Supplementary file 1 — Expanded View Figures PDF [file EMBJ-42-e113018-s001.pdf]

Expanded View Figures

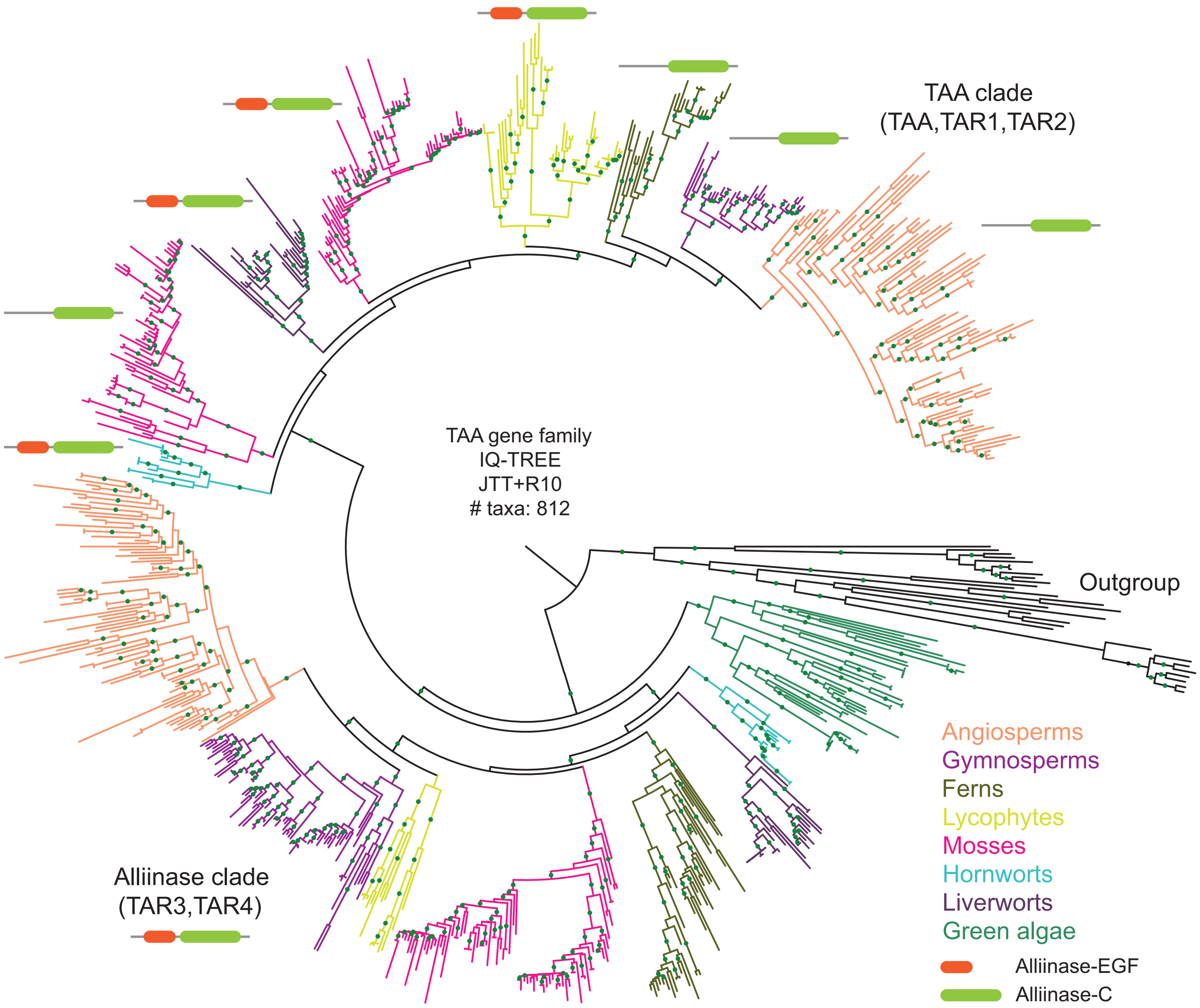

Figure EV1. Phylogenetic tree of the TAA gene family with green algae and land plant homologs.

Protein domains, Alliinase-EGF and Alliinase-C are indicated with "red" and "green" representations, respectively. TAR3/TAR4 clade shows the consistent presence of both domains in all lineages, whereas in TAA/TAR1/TAR2 clade, some phyla lack Alliinase-EGF domain. Aminotransferases from land plants, other than TAA members, were used as outgroup sequences to root the tree. Branches that are well supported (bootstrap > 75) are indicated by green dots. Orthologs from each phylum are represented in different colors, as indicated in the right bottom right legend. Basic information about the tree construction: "software," "model of evolution," and the "number of taxa" used for phylogenetic tree construction are indicated at the center. The complete tree can be found at the interactive Tree of Life (iTOL) repository: https://itol.embl.de/shared/dolfweijers.

© 2023 The Authors

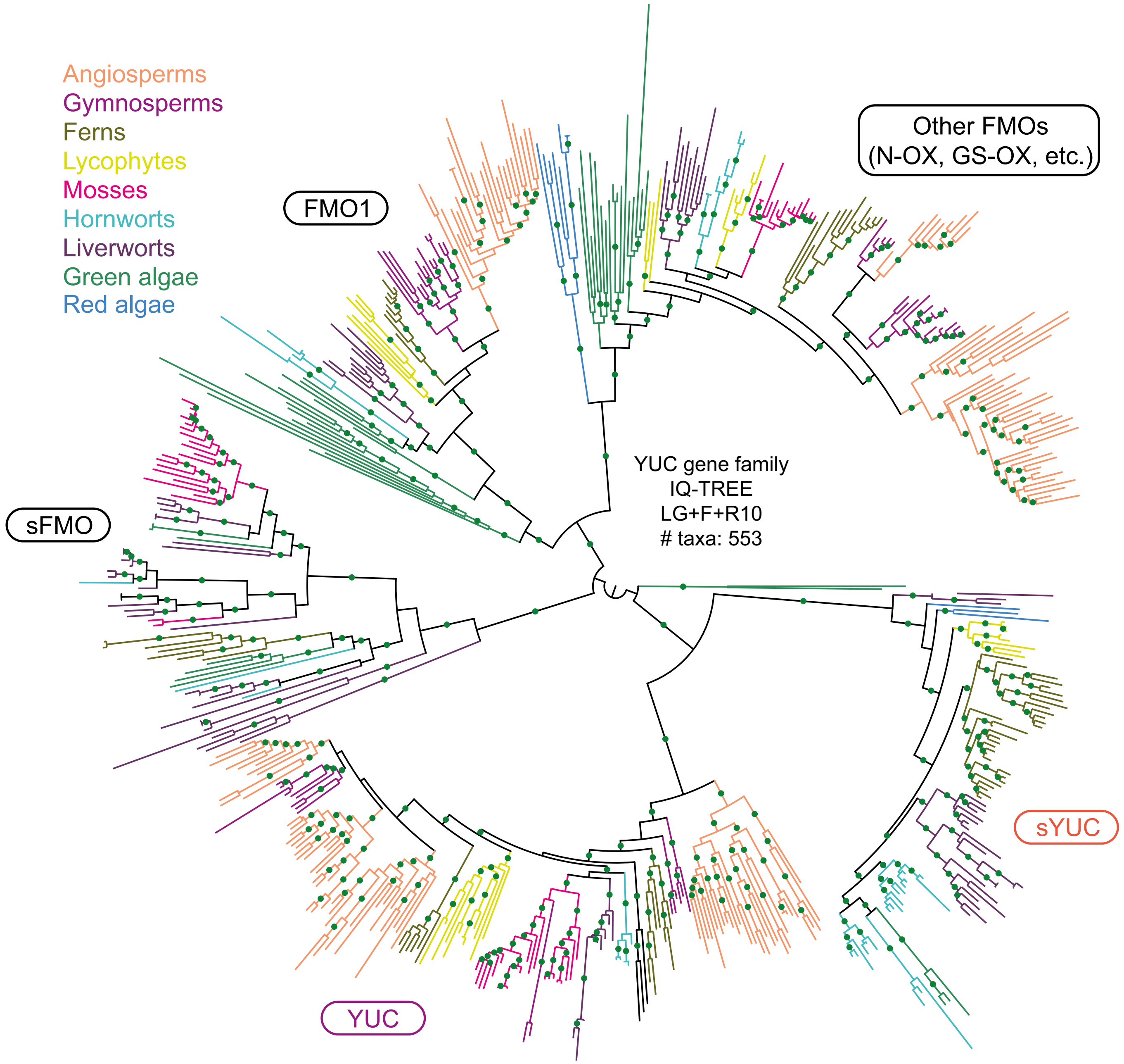

Figure EV2. Phylogenetic tree of the YUC gene family with algae and land plant homologs.

The different FMO clades are indicated by representative *Arabidopsis* family member names (FMO, flavin monooxyenase 1; GS-OX, FMO glucosinolate S-oxygenase; N-OX, FMO N-oxygenases; YUC, Yucca). Branches that are well supported (bootstrap > 75) are indicated by green dots. Orthologs from each phylum are represented in different colors, as indicated in the bottom right legend. Basic information about the tree construction: "software," "model of evolution," and the "number of taxa" used for phylogenetic tree construction are indicated at the center. The complete tree can be found at the interactive Tree of Life (iTOL) repository: https://itol.embl.de/shared/dolfweijers.

 © 2023 The Authors

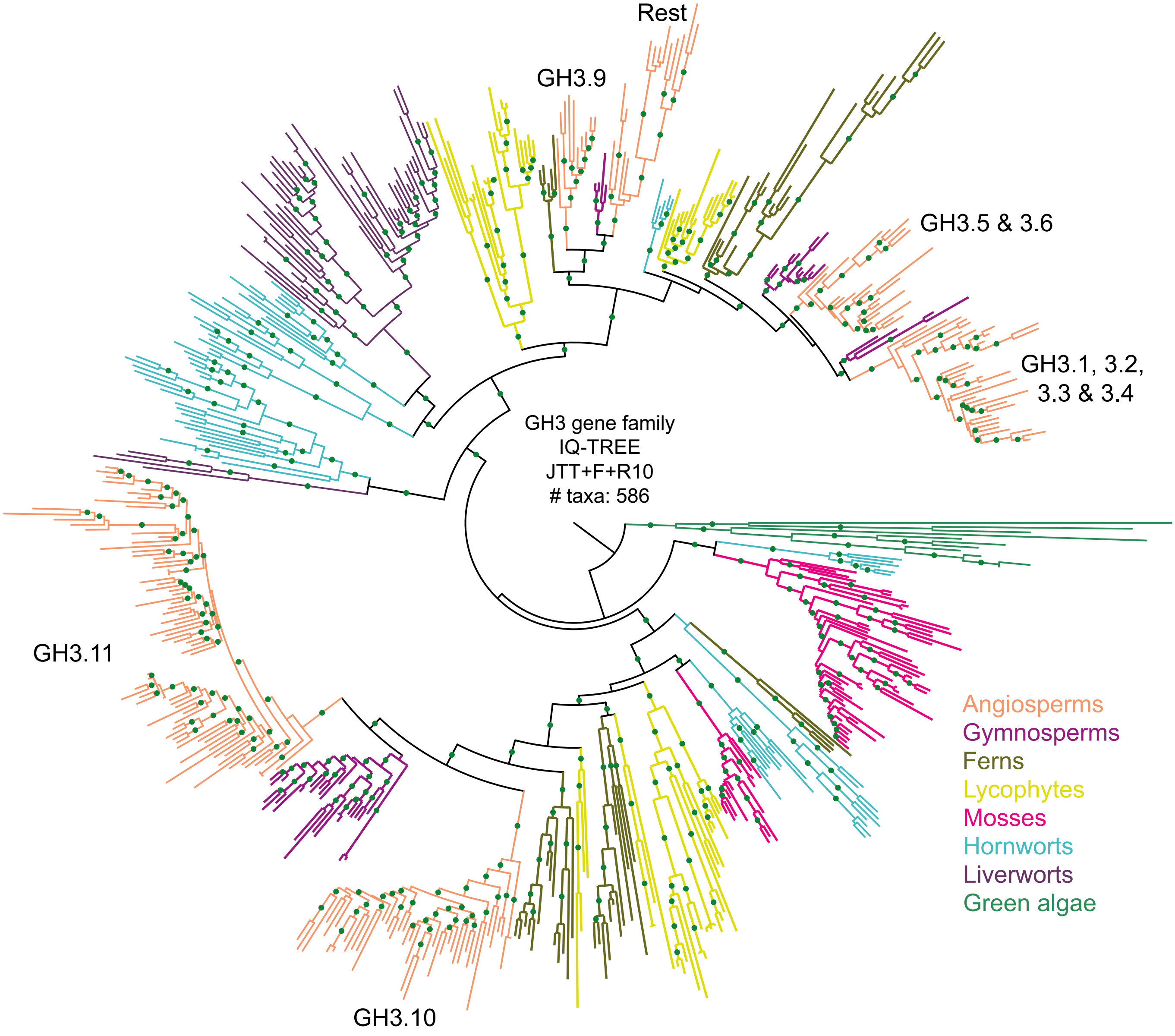

Figure EV3. Phylogenetic tree of the GH3 gene family with green algae and land plant homologs.

Respective *Arabidopsis* orthologs that are present in the specific clade are mentioned with the corresponding *Arabidopsis* family member names. "Rest" includes GH3.7, 3.8, 3.12 until 3.19. Branches that are well supported (bootstrap > 75) are indicated by green dots. Orthologs from each phylum are represented in different colors, as indicated in the bottom right legend. Basic information about the tree construction: "software," "model of evolution," and the "number of taxa" used for phylogenetic tree construction are indicated at the center. The complete tree can be found at the interactive Tree of Life (iTOL) repository: https://itol.embl.de/shared/dolfweijers.
